# Manganese-Substituted Polyoxometalates as Functional Mimics of Indole Dioxygenase Enzymes

**DOI:** 10.1021/acs.inorgchem.6c02005

**Published:** 2026-06-01

**Authors:** Adi Herman, Raanan Carmieli, Ronny Neumann

**Affiliations:** † Department of Molecular Chemistry and Materials Science, 34976 Weizmann Institute of Science, Rehovot 76100, Israel; ‡ Department of Chemical Research Support, Weizmann Institute of Science, Rehovot 76100, Israel

## Abstract

Dioxygenase reactivity, where both oxygen atoms of O_2_ are incorporated into an organic product, is a relative rarity. Notable is the indoleamine 2,3-dioxygenase enzyme with an iron-heme active site. Biomimetic studies using iron and manganese porphyrins have been successful and mechanistically enriching. We show that inorganic manganese-substituted polyoxometalates are also effective functional mimics of indoleamine 2,3-dioxygenases. Thus, several mono-, di-, and tri-Mn^II^-substituted polyoxometalates were reacted with O_2_, and the intermediacy of a superoxide species was supported by both EPR spectroscopy using 5-*tert*-butoxycarbonyl-5-methyl-1-pyrroline-*N*-oxide as a spin trap and cyclic voltammetry. Further experiments with 2,3-dimethyl-1*H*-indole yielded the expected dioxygenated product, *N*-(2-acetylphenyl)­acetamide, in high yields under O_2_. Oxygen isotope labeling experiments and the use of triphenylphosphine as a probe supported a stepwise oxygenation process with formation of two suggested manganese-centered intermediates as also shown for porphyrin/heme-based catalysts. Using a combination of UV–vis and EPR measurements, it was concluded that after a first oxygen donation by a Mn­(III)-superoxo species, a second oxygen-donated species, best formulated as a Mn­(III)-oxyl species rather than a Mn­(IV)-oxo species, yielded the final dioxygenated product. The research supports the notion that inorganic manganese polyoxometalates with an oxotungstate framework are true functional models of indoleamine 2,3-dioxygenases.

## Graphical abstract

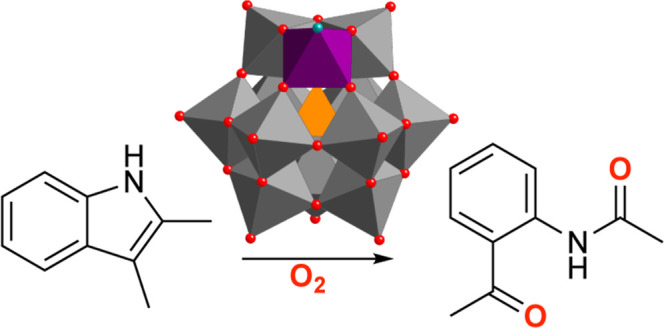

## Introduction

Research on bioinspired molecular oxygen activation at metal centers can be divided to those having monooxygenase type activity, where one oxygen atom of O_2_ yields H_2_O and dioxygenase type activity where both oxygen atoms of O_2_ are incorporated into the product. Iron-based dioxygenases can be divided into three types: Monoiron nonheme enzymes such as catechol dioxygenases;^1,2^ the Reiske type dioxygenase that catalyzes the *cis*-dihydroxylation of arenes in the presence of NADH as a reducing agent;^1,2^ and enzymes with iron-heme active sites, indoleamine 2,3-dioxygenase (IDO), and tryptophan 2,3-dioxygenase (TDO).^3^ The mechanism of the IDO and TDO enzymes have been studied,^4–6^ and IDO activity has also been studied using biomimetic iron porphyrin catalysts,^7–9^ and further extended to manganese porphyrins, as well as other functional biomimetic models.^10–12^

The activation of molecular oxygen with polyoxometalates is a rather broad topic that includes inner-sphere four-electron reduction to water using phosphovanadomolybdates, autoxidations and reactions with sacrificial reductants, and outer-sphere electron transfer from polyoxotungstates to dioxygen.^13^ Typical transformations resulting from these types of activation of molecular oxygen include oxidative dehydrogenations and oxygenations via radical-propagated mechanisms. More recently, iron- and copper-substituted polyoxometalates have been shown to undergo monooxygenase-type reactions reminiscent of iron porphyrins, including the recent electroreductive activation of O_2_ toward alkane hydroxylation catalyzed by iron- and copper-substituted polyoxometalates.^14–16^ Dioxygenase activity using ruthenium-substituted polyoxometalates was also reported, albeit reactions were not generally catalytic except for very active substrates such as *trans*-cyclooctene.^17,18^

Continuing our interest in investigating the use of inorganic polyoxometalates in reactivity functionally similar to those of metalloenzymes and related biomimetic compounds, we report on the use of manganese- and iron-substituted polyoxometalates as functional mimics of indole dioxygenase enzymes. The research is based mostly on Keggin type compounds where monolacunary K_8_[α-SiW_11_O_39_]·13H_2_O is substituted with M = Mn^II^ to yield {α-SiMn^II^(H_2_O)­W_11_O_39_}^6–^ anions, and trilacunary Na_9_[β-SiW_9_O_34_H]·23H_2_O is substituted M = Mn^II^, Zn^II^ to yield homo- (Mn^II^) and hetero-substituted (Mn^II^, Zn^II^) {α-SiM^II^_3_(H_2_O)_3_W_9_O_37_}^10–^ anions.^19–21^ The research hypothesis was based on two possible scenarios for indoleamine 2,3-dioxygenase type activation of O_2_, Figure 1.

**Figure 1:**
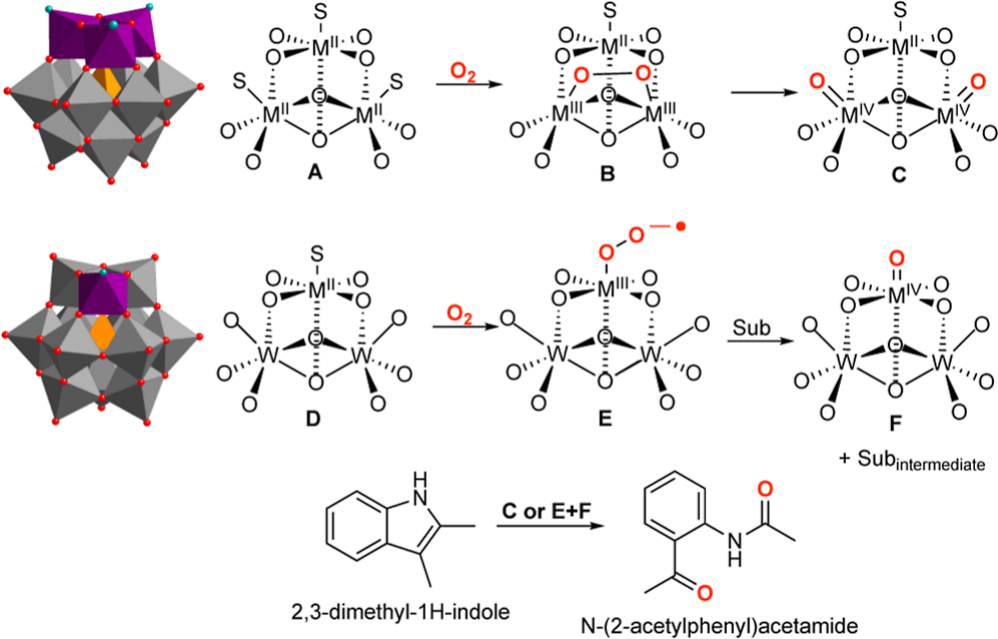
Possible generic scenarios for molecular oxygen activation for the dioxygenation of 2,3-methyl 1*H*-indole to *N*-(2-acetylphenyl)­acetamide. Top pathway - intramolecular activation of O2 at two adjacent M^II^ = Mn, Zn, or Fe sites; bottom pathway −O_2_ activation at a single M^II^ site. Gray–W; orange–Si, red–O, and light blue −H_2_O. Other possibilities include two end-on superoxo species instead of species B and also side-on superoxo species as an alternative to compound E.

The first pathway posited an intramolecular activation of O_2_ at two adjacent metal sites involving formation of two M^IV^ = O oxo or Mn­(III)-oxyl species (Figure 1, A → B → C) from two Mn^II^ centers, followed by oxygenation from equivalent or at least very similar Mn^IV^ = O/Mn­(III)-oxyl intermediate species. The second pathway, requiring only a single reactive site, proposed a formation of a M^III^-O_2_^•–^ superoxide intermediate (Figure 1, E) as an active species for IDO activity, with co-formation of a M^IV^ = O/Mn­(III)-oxyl species (Figure 1, F) for the further formation of the final product. As will be shown, the research indicates IDO reactivity at a single metal reaction site by the second pathway.

## Results and Discussion

Chronologically speaking, the research was begun to test the possibility of any intramolecular activation of O_2_ at two adjacent metal sites involving the formation of two Mn^IV^ = O oxo species (Figure 1 A → B → C) since such intramolecular activations are rare and have not been documented for polyoxometalates. Thus, tetrahexylammonium (THA) salts of {α-SiMn^II^_3‑x_Zn^II^_*x*_(H_2_O)_3_W_9_O_37_}^10–^ (*x* = 0, 1, 2) and {α-SiFe^II^_3_(H_2_O)_3_W_9_O_37_}^10–^ (*x* = 0, 1, 2) were prepared according to literature procedures.^19,21^ The characterization of these compounds was by infrared (IR) and high-resolution electrospray ionization mass spectrometry (HR-ESI–MS) for the newly synthesized hetero-substituted (Mn^II^, Zn^II^) anions, as detailed in the experimental section and the Supporting Information

To examine the reaction of the different manganese compounds with molecular oxygen, EPR spectroscopy was used to detect oxygen-centered radicals through the formation of spin adducts. First solutions of [SiMn_3_(L_3_)­W_9_O_37_], {SiMn_3_W_9_}, in THF or acetonitrile (ACN) in the presence of 5-*tert*-butoxycarbonyl-5-methyl-1-pyrroline-*N*-oxide (BMPO) as a spin trap were exposed to O_2_. EPR spectra were measured before and after the addition of O_2_, as shown in Figure 2.

**Figure 2:**
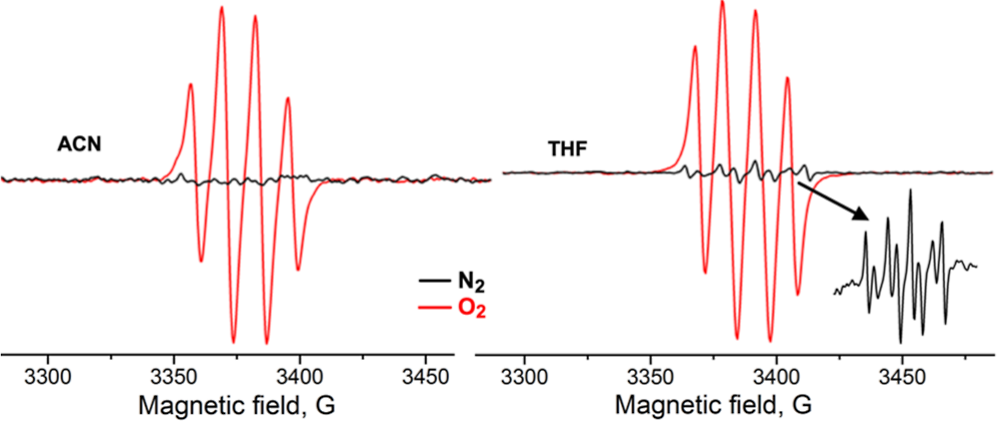
EPR spectra of {SiMn_3_W_9_} in ACN (left) and THF (right) under N_2_ (black) and after (red) exposure to O_2_ for 50 min in the presence of BMPO. Conditions: 2 mM {SiMn_3_W_9_}, 5 mg BMPO, 1.5 bar O_2_ at room temperature.

Under nitrogen in ACN, no formation of a BMPO spin adduct is observed, but in THF, a relatively low intensity spectrum is observed. This spectrum (see insert in Figure 2) is associable to a BMPO-carbon radical adduct that in turn can be assigned to an electron transfer between {SiMn_3_W_9_} and a coordinated THF molecule to a Mn atom. Such THF ligation to an Fe atom has been previously observed by computation for analogous {SiFe_3_W_9_}.^22^ After treatment of {SiMn_3_W_9_} with O_2_ in both ACN and THF, spectra clearly associated with the hydroxy spin adduct, BMPO/OH^•^, are obtained.^23^ The formation of BMPO/OH^•^ could be associated with the formation of a hydroxyl radical or a Mn^III^_2_-peroxo or a reactive Mn-oxo intermediate species (Figure 1, B and C) as originally posited. However, studies with a very nucleophilic substrate such as triphenylphosphine (TPP), presumably reactive toward Mn^III^_2_-peroxo or Mn^IV^ = O intermediate species, yielded no product (see also below). Alternatively, it should be noted that superoxo spin adducts such as BMPO/OOH^•^ have a limited lifetime, leading to the formation of BMPO/OH^•^ and that such a transformation can be catalyzed by {SiMn_3_W_9_}.

To explore such a BMPO/OOH^•^ → BMPO/OH^•^ transformation versus direct reaction of BMPO with a hydroxyl radical, spin trapping experiments were repeated in the presence of DMSO. Since hydroxyl radicals react faster with DMSO than with BMPO and form methyl radicals,^24–26^ the absence of a change in intensity in the EPR spectrum upon addition of DMSO and the absence of a BMPO/CH_3_^•^ would support the initial formation of BMPO/OOH. This is indeed what is observed, Figure S1. While this experiment precludes the formation of a hydroxyl radical, it does not rule out the formation of Mn^III^_2_-peroxo or Mn^IV^ = O intermediate species. To do so, mono-Mn-substituted polyoxometalates, such as THA_6_[SiMn^II^(L)­W_11_O_39_], {SiMnW_11_}, and THA_8_[α_2_-P_2_Mn^II^(L)­W_17_O_61_], {P_2_MnW_17_}, were reacted with O_2_ in the presence of BMPO. Such reactions have been reported in the past, indicating the reversible formation of Mn^II^–O_2_ species at room temperature.^27^

In Figure 3, one can see that the addition of O_2_ to {SiMnW_11_}/BMPO shows a spectrum with elements of both BMPO/OOH^•^ and BMPO/OH^•^. Somewhat differently, the addition of O_2_ to {P_2_MnW_17_}/BMPO showed a spectrum of BMPO/OH^•^ only, while a spectrum carried out in the presence of air showed a low intensity spectrum of BMPO/OOH^•^. These experiments clarify the intermediacy of a superoxo species and the fast transformation of BMPO/OOH^•^ → BMPO/OH^•^ in the absence of an IDO substrate under experimental conditions.

**Figure 3:**
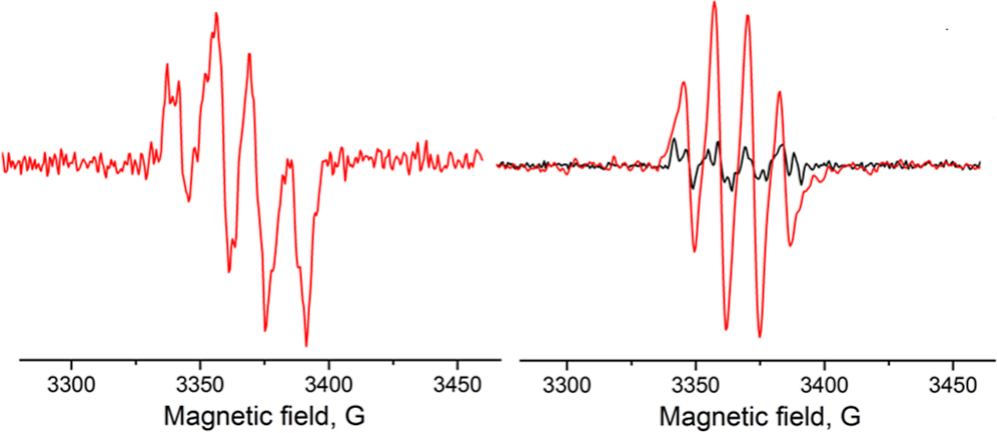
EPR spectra of {SiMnW_11_} (left) and {P_2_MnW_17_} (right) in the presence of BMPO. Conditions: 2 mM polyoxometalate, 5 mg BMPO in acetonitrile, under air (black) and after (red) exposure to 1.5 bar O_2_ for 1 h in the presence of BMPO at room temperature.

The EPR measurement of K_6_{SiMnW_11_} in water gave the typical 6- line EPR spectrum at *g* = 2 for an octahedral Mn­(II) compound, Figure 4 (left), with 5 oxygen ligands from the polyoxometalate framework and one labile terminal water ligand. However, the EPR spectrum of THA_6_{SiMnW_11_} in ACN showed a six-line spectrum at g ∼4 associated with a five-coordinate square pyramidal Mn­(II) compound where the labile oxygen ligand is absent, as also previously observed for another Mn­(II)-substituted polyoxometalate, Figure 4 (right).^28^ The vacant coordination site renders the formation of a Mn­(II)–O_2_ ↔ Mn^III^–O_2_^•^– very plausible.

**Figure 4:**
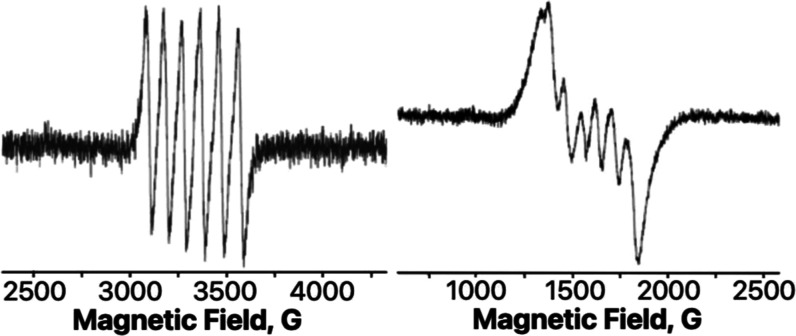
EPR spectra of K_6_{SiMnW_11_} under N_2_ in water at 298 K (left) and THA_6_{SiMnW_11_} under N_2_ in ACN at 120 K (right).

Although direct observation of a Mn^III^–O_2_^•^– species was not successful at low temperature, such a species was observable for an analogous iron compound, {SiFe_3_W_9_}, Thus, under air in THF, {SiFe_3_W_11_}, yielded a EPR spectrum consisting of a Fe^III^ signal at g = ∼4, Figure S2, and a less intense signal assignable to a Fe^III^–O_2_^•^– species,^29^ Figure 5. Reactions in the presence of BMPO gave the spectrum typical for a BMPO/OH^•^ specie, Figure S3, as discussed above.

**Figure 5:**
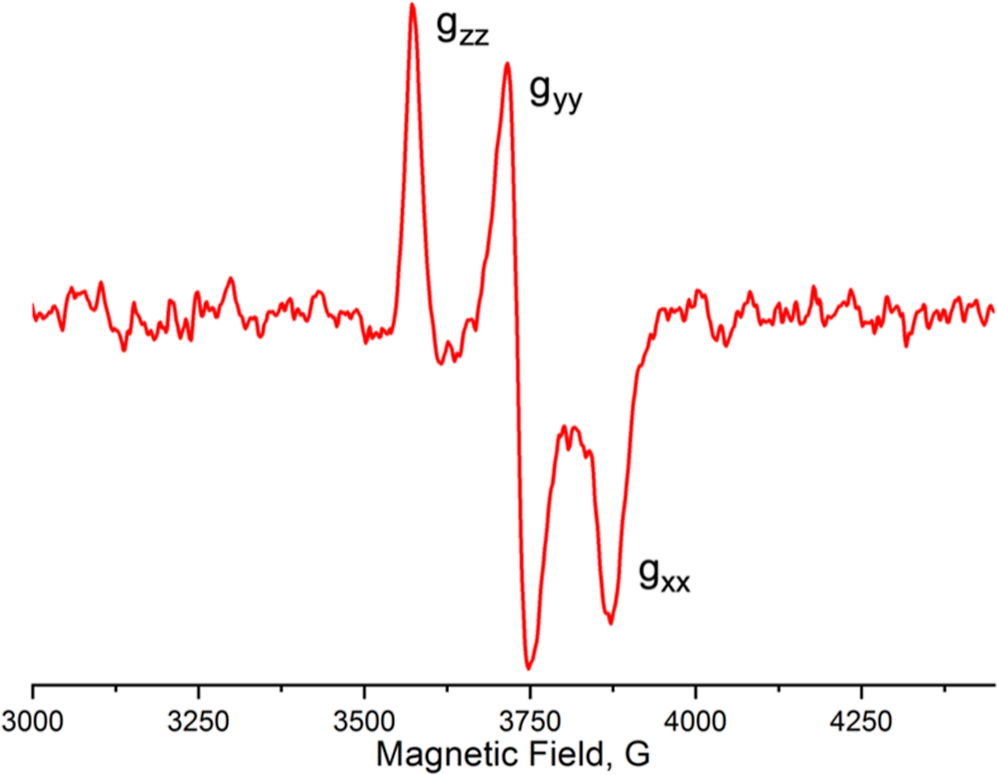
EPR spectrum of 2 mM THA_6_[SiFe^II^(L)­W_11_O_39_] in THF at 15 K after exposure to air.

Another way to examine the interaction of polyoxometalates with O_2_ is by cyclic voltammetry (CV). Figure S4 shows the CV of O_2_ with the typical reversible redox transformation, O_2_ ↔ O_2_^•^ at −1.285 V versus Fc/Fc^+^. In the presence of {SiMn_3_W_9_}, Figure 6, the CV is unchanged indicating a reversible {SiMn_3_W_9_} + O_2_ ↔ {SiMn_3_W_9_}^+^-O_2_^•^– transformation as previously suggested.^27^ Upon the addition of ethanol as a proton donor, the reduction of O_2_ becomes irreversible, supporting the formation of an unstable protonated superoxo species, {SiMn_3_W_9_}^+^-O_2_H that decomposes.

**Figure 6:**
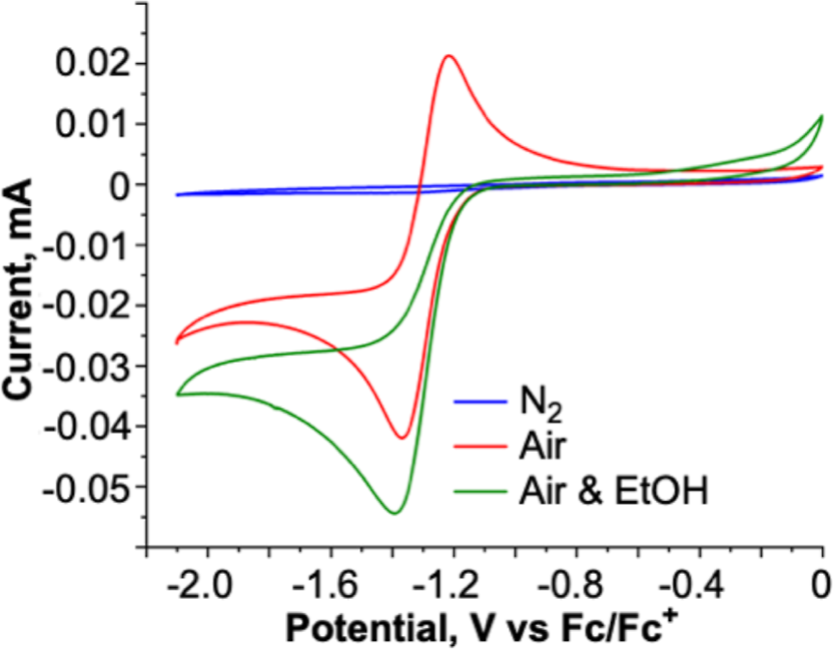
CV of (THA)_10_[SiMn_3_(L_3_)­W_9_O_37_] in ACN under N_2_ or air, with and without 50 μL ethanol. Conditions: 2 mM POM, 0.1 M TBABF_4_ – 5 mL CAN. Working electrode - glassy carbon; counter electrode - Pt wire; reference electrode −Fc/Fc^+^; and scan rate −50 mV/s.

Inspired by the activity of TDO and IDO enzymes, the oxidation of 2,3-dimethylindole (2,3-DMI) was examined under the presence of air and then under oxygen, Figure 7. The reactions with 2,3-DMI gave 3 products, Scheme 1. The main product is the dioxygenated product, *N*-(2-acetylphenyl)­acetamide. Two additional products are mono-oxygenated products with molecular peaks in their mass spectra at *m*/*z* 161. These products, 3,3-dimethylindolin-2-one and 2,2-dimethylindolin-3-one, are suggested to be the result of the rearrangement of the intermediate epoxide that was previously observed in the oxidation of the nitrogen atom-protected analog, *N*-acetyl-2,3-dimethylindole.^30^ The products were identified by GC–MS and quantified by GC-FID, Figures S5 and S6. The activity of the {SiFe_3_W_11_} compound was also tested but was significantly less reactive, yielding only ∼6 mol % product within 20 h.

**Figure 7:**
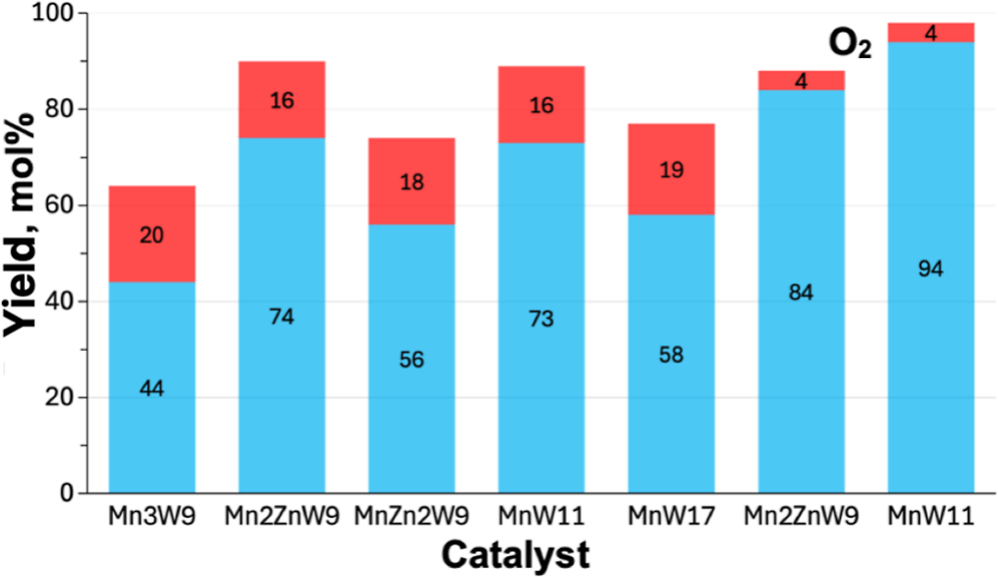
Oxygenation of 2,3-DMI with different polyoxometalates under 1 bar air or O_2_ (two right columns). Conditions 2 mM polyoxometalate, 33 mM 2,3-DMI, 50 μL EtOH in 5 mL ACN, *t* = 24 h. Turquiose - *N*-(2-acetylphenyl)­acetamide; red–monooxygenates −3,3-dimethylindolin-2-one and 3-methyl-2-methyleneindolin-3-ol.

**Scheme 1:**
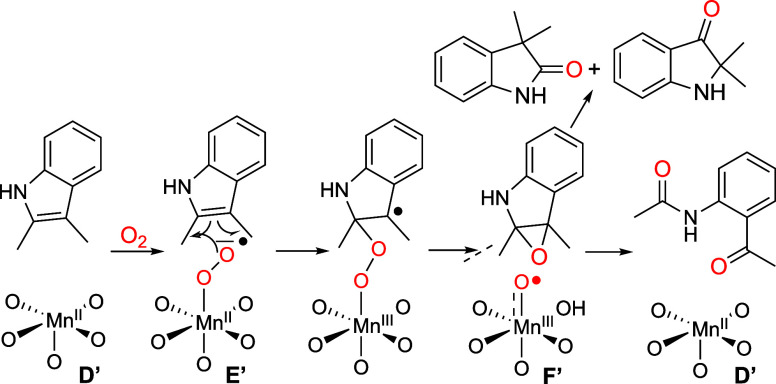
Product Formation through a Proposed Epoxide Intermediate

The overall yields of the reactions in air were typically between 75 and 90% with the dioxygenated *N*-(2-acetylphenyl)­acetamide as the major product. The yield with {SiMn_3_W_9_} was somewhat lower. Clearly, monomanganese-substituted catalysts are reactive. The higher activity of {SiMnW_11_} versus {SiMnZn_2_W_9_} and {P_2_MnW_17_} may be related to the overall higher negative charge of the latter compounds, resulting in some electrostatic substrate repulsion, as also previously observed in other O_2_-based reactions.^16^ Poly manganese-substituted polyoxometalates are also reactive, where the higher reactivity of {SiMn_2_ZnW_9_} versus {SiMn_3_W_9_} may be related to a stabilization of the superoxo intermediate by the Lewis acidic Zn center.^21,31^ This stabilization may also be *a* factor in the higher activity of {SiMnZn_2_W_9_} versus {P_2_MnW_17_} that have the same 8– charge. Under 1 bar O_2,_ both the yields and the selectivity to the dioxygenated *N*-(2-acetylphenyl)­acetamide major product are significantly increased.

To identify the source of the oxygen atoms in the dioxygenated product, an oxygenation reaction with labeled ^18^O_2_ was performed. By using GC–MS, Figure S7, we observed that both oxygen atoms in *N*-(2-acetylphenyl)­acetamide were ^18^O labeled supporting a dioxygenase pathway. However, as proposed in Scheme 1, the formation of an epoxide intermediate, a further reactive Mn-oxo intermediate, and some formation by rearrangement to monooxygenated products, Figure 6, suggests that the two oxygen atoms in *N*-(2-acetylphenyl)­acetamide product do not necessarily come from the same original O_2_ molecule. In support of a stepwise reaction, a mixture of ^18^O_2_/^16^O_2_ with a quantified ratio by GCMS *m*/*z* 36/32 = 1.64, was reacted under the conditions in Figure 6. The GC–MS, Figure S8, shows the prominence of the mixed labeled ^18^O^16^O *N*-(2-acetylphenyl)­acetamide (*m*/*z* = 179) versus the monolabeled *N*-(2-acetylphenyl)­acetamide (*m*/*z* 177 and 181) that supports a sequential reaction mechanism.

In previous reports on the mechanism of IDO/TDO, there appears to be a consensus that the second oxygenation reaction occurs through a ferryl-oxo species.^6,8,9^ By analogy, one could posit that similarly, a Mn­(IV)–O intermediate would be formed, which was also proposed for a Mn-heme catalyzed reaction.^12^ The formation of such an oxygen donor intermediate was initially inferred by adding the very reactive nucleophilic triphenylphosphine (TPP) to the reaction mixture. Thus, 2 mM {SiMnW_11_}, 36 mM 2,3-DMI, 36 mM TPP, and 50 μL EtOH in 2 mL ACN were reacted for 24 h under 1.5 bar O_2_. Both the IDO product, *N*-(2-acetylphenyl)­acetamide, and triphenylphosphine oxide (TPPO), 25 μmol and 55 μmol, respectively, were formed. Importantly, a control experiment without 2,3-DMI yielded only traces of TPPO (2.5 μmol), showing that a Mn-oxo intermediate species is formed after the first oxygen transfer to 2,3-DMI as proposed in Scheme 1 (E′ and F′ intermediates). A further control experiment in a reaction of 2,3-DMI and TPP without a catalyst also showed only traces of TPPO formation.

The formation of a proposed Mn-oxo intermediate was observed by UV–vis measurements. The UV–vis spectrum of {SiMnW_11_} prepared under N_2_ is noticeably different from the analogous spectrum upon the addition of O_2_, Figure S9. The change is assignable to the formation of a Mn­(III)-superoxo species. Upon addition of 2,3-DMI, the formation of a further intermediate species λ_max_ = 475 nm is observable with an isosbestic point at λ = 500 nm, Figure 8.

**Figure 8:**
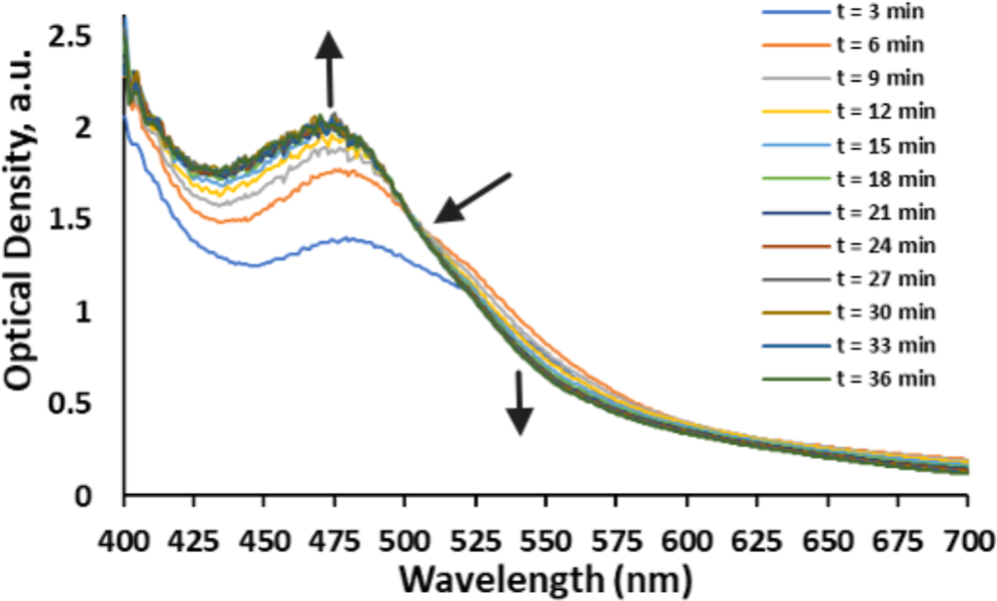
UV–vis spectra of 3 mM {SiMnW_11_} in ACN and O_2_ upon addition of 10 mM 2,3-DMI.

While it is enticing to assign this new species as a Mn­(IV)-oxo compound as suggested for heme-based catalysts, it should be noted that previous studies have shown that Mn­(IV) substituted into a polyoxometalate are quite basic leading to formation of Mn­(IV)-hydroxide compounds that can be isolated and are not reactive oxygen donors even to TPP.^32,33^ Thus, in an EPR experiment designed to observe the intermediate species (F′, Scheme 1), 33 mM 2,3-DMI was added to a solution of 10 mM {SiMnW_11_} in acetonitrile under N_2_. The solution was cooled to 273 K and treated with O_2_ for 10 min to initiate a dioxygenation reaction and then frozen with liquid nitrogen. The EPR spectrum at 120 K obtained, Figure 9, shows both a low spin Mn­(III) species that can be attributed to a Mn­(III)-oxyl intermediate (Mn^III^–O_2_^•^– species was not observed by EPR at 120 K) and the initial Mn­(II) compound D′ formed after dioxygenation.

**Figure 9:**
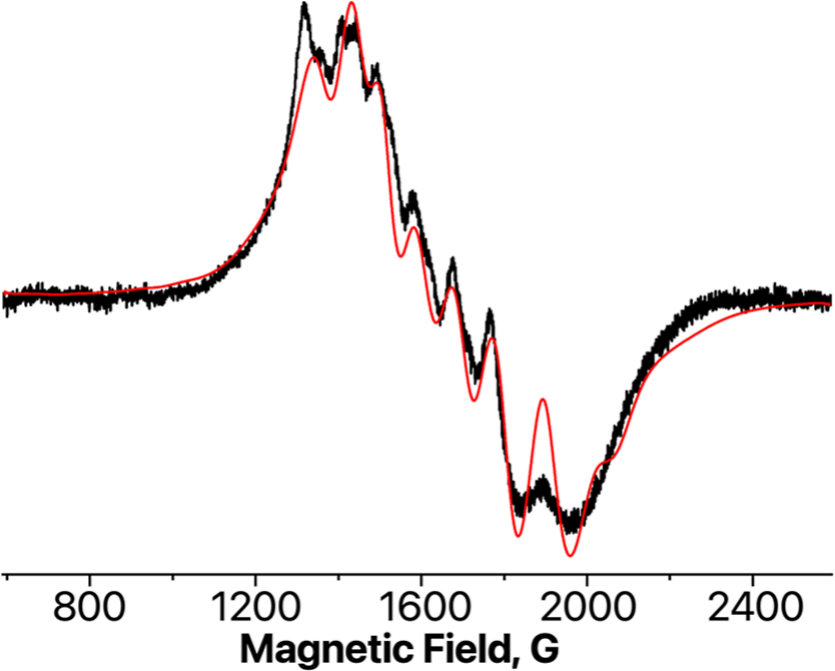
EPR spectrum (black) and simulated spectrum (red) of {SiMnW_11_} in ACN at 120 K. Simulation parameters: (1) Mn­(III) S = 1, g_1_ = 4.21, A_1_ = 510 MHz, Iw_1_ = 3.8 mT, D_1_ = 250 MHz, D strain = 420 MHz; (2) Mn­(II), S = 5/2, g_2_ = 3.92, A_2_ = 510 MHz, Iw_2_ = 2.8 mT, D_2_ = 700 MHz, D strain = 420 MHz. Attempts at simulating the spectrum with Mn­(III) S = 2 or Mn­(IV) failed.

## Conclusions

The substitution of Mn^II^ and Fe^II^ cations into lacunary positions of polyoxotungstates led to species that upon reaction with O_2_ led to the initial formation of superoxo species, Mn^III^–O_2_^–•^ or Fe^III^–O_2_^–•^. For Mn^III^–O_2_^–•^, the assignment was supported by EPR using 5-*tert*-butoxycarbonyl-5-methyl-1-pyrroline-*N*-oxide as a spin trap. The intermediacy of a hydroxyl radical was ruled out by the addition of DMSO as a radical trap. Using 2,3-dimethylindole as a substrate, the formation of *N*-(2-acetylphenyl)­acetamide via a dioxygenase reaction was carried out. In the presence of 1 bar O_2_, the reaction was almost quantitative and mostly selective. Minor amounts of monooxygenated compounds were also obtained and identified. Reaction yields were negatively affected by higher anionic charges of the polyoxometalate catalyst but positively affected by the presence of Lewis acidic Zn­(II) that can stabilize intermediate superoxo species. Use of labeled ^18^O_2_ supported the dioxygenase pathway with a sequential reaction pathway of successive oxygen transfer reactions. An overview of the entire reaction mechanism as supported by various UV–vis, CV, and EPR experiments presented in Scheme 1 indicates that a Mn^III^–O_2_^–•^ species is the first oxygen donor species, while the second oxygen donor species points to a Mn­(III)-oxyl intermediate as a most likely conclusion. The research supports the finding that transition metal substituted into polyoxometalate frameworks are surprising functional mimics of heme oxygenases as also previously observed for polyoxometalates showing monooxygenase activity.^14–16^

## Experimental Part

### Polyoxometalate Syntheses

Lacunary POMs K_8_[α-SiW_11_O_39_]·13H_2_O, Na_9_[β-SiW_9_O_34_H]·23H_2_O, and K_10_[α_2_-P_2_W_17_O_61_]·20H_2_O were synthesized according to the known literature procedures.^34^ K_6_[α-SiMn­(H_2_O)­W_11_O_39_] was synthesized by reacting manganese sulfate, MnSO_4_·H_2_O, with K_8_[α-SiW_11_O_39_]·13H_2_O according to a known literature procedure.^35^ K_10_[α-Si­{Mn­(H_2_O)}_3_W_9_O_37_] was synthesized by reacting manganese acetate, Mn­(CH_3_CO_2_)_2_·4H_2_O, with Na_10_[α-SiW_9_O_34_] according to a known literature procedure.^36^ K_8_[α_2_-P_2_ Mn^II^(H_2_O)]­W_17_ O_61_} was synthesized by reacting manganese chloride, MnCl_2_·4H_2_O, with K_10_[α_2_-P_2_W_17_O_61_] according to a known literature procedure.^37^ Cs_10_[β-SiW_9_O_37_{Mn_2_Zn­(H_2_O)}_3_] and Cs_10_[β-SiW_9_O_37_{MnZn_2_(H_2_O)}_3_] were synthesized by reacting “triple” salts, [Mn_2_ZnO­(MeCO_2_)_6_(H_2_O)_3_] and [MnZn_2_O­(MeCO_2_)_6_(H_2_O)_3_], with Na_9_[β-SiW_9_O_34_H]·23H_2_O as previously outlined in the literature.^20^ Thus, [Mn_2_ZnO­(MeCO_2_)_6_(H_2_O)_3_] and [MnZn_2_O­(MeCO_2_)_6_(H_2_O)_3_] were prepared by adding a solution of NaOAc·H_2_O (0.31 mol) in water (70 mL) to a filtered, stirred solution of manganese nitrate (0.02 or 0.01 mol) and zinc nitrate (0.01 or 0.02 mol) in water (70 mL). This resulted in a colored solution, which was evaporated and dried under vacuum. Na_9_[β-SiW_9_O_34_H]·23H_2_O (1.5 mmol) was added in small amounts with vigorous stirring to a solution of [Mn_2_ZnO­(MeCO_2_)_6_(H_2_O)_3_] or [MnZn_2_O­(MeCO_2_)_6_(H_2_O)_3_] (11.7 mM) in NaOAc (pH 6.5, 150 mL, 0.25 M), and the mixture was then heated to 50 °C for 1 h. The colored solution that was formed was cooled and treated with a solution of CsCl (0.33 g/mL) until the formation of precipitates of Cs_10_[β-SiW_9_O_37_{Mn_2_Zn­(H_2_O)}_3_] and Cs_10_[β-SiW_9_O_37_{MnZn_2_(H_2_O)}_3_]. These new compounds were characterized by high resolution electrospray ionization mass spectrometry and IR spectrometry as previously reported, Figures S10 and S11.^20^ Metathetical exchange of the alkali cations with tetrahexylammonium (THA) was carried out in dichloromethane or toluene using stoichiometric amounts of tetrahexylammonium bromide (THABr) to synthesize polyoxometalates soluble in organic solvents. Unless otherwise noted, the polyoxometalates were used as THA compounds.

### Electrochemistry

All experiments were carried out using a BioLogic Science VSP-201 potentiostat. CV was performed in an 18 mL airtight Pyrex vial with a 5 mL solution. The conditions were 2 mM (THA)_10_[SiMn_3_(L_3_)­W_9_O_37_] 0.1 M tetrabutylammonium tetrafluoroborate (TBABF_4_) as a supporting electrolyte, a glassy carbon disk as a working electrode, Pt wire as a counter electrode, and Fc/Fc + as a reference electrode. All solutions were prepared under N_2_ in a glovebox, and the experiments were performed under a N_2_ or air/O_2_ mixture.

### Product Analysis

Combined GC-FID and GC-MSD measurements were carried out to quantify and identify reaction liquid phase products, respectively, using a HP 6890 instrument with a flame ionization detector and a HP 5973 instrument with a mass selective detector. The separations were carried out using a 30 m column (Restek 5 MS, 0.32 mm internal diameter) that has a 5% phenylmethyl silicone coating of 0.25 μm; helium was the carrier gas. Product identification was also verified using ACD/MS Fragmenter with Spectrus Processor Software.

### EPR Measurements

EPR measurements were carried out at room temperature by using a Bruker ELEXYS E500 spectrometer operating at X-band frequencies (9.5 GHz) and a Bruker ER4119HS resonator. Samples were loaded into Vitrocom quartz capillaries, CV1012-Q-100, with a 1 mm inner diameter. The experimental conditions of the EPR measurements were as follows: microwave power of 20 mW, 1 G modulation amplitude, and 100 kHz modulation frequency. For measurements with BMPO, the sweep range was 200 gauss and the spectra consisted of 400 points. For manganese measurement, the sweep range was 1500 G and the spectra consisted of 750 points.

Low-temperature CW EPR measurements were carried out at 15 K or 120 K on a Bruker Elexsys E580 spectrometer operating at 9.5 GHz and outfitted with an X-band resonator (EN-4118X-MD4). The temperature was controlled by an Oxford Instruments CF935 continuous-flow cryostat using liquid He or N_2_. The experimental conditions of the EPR measurements were as follows: microwave power of 20 mW, 1 G modulation amplitude, and 100 kHz modulation frequency. 2000 G and the spectra consisted of 1000 points.

## Supplementary Material



## Notes

Additional EPR spectra; UV–vis spectra, CV scans; GC-FID chromatograms; mass spectra from GC-MSD chromatograms; peaks from HR-ESI-MS of new compounds; IR spectra of new compounds (PDF)

## Abbreviations:

ACN: acetonitrile
BMPO: 5-*tert*-butoxycarbonyl-5-methyl-1-pyrroline-*N*-oxide
2,3-DMI: 2,3-dimethyl indole
IDO: indole dioxygenase
TDO: tryptophan dioxygenase
THA: tetrahexylammonium
TPP: triphenyl phosphine
TPPO: triphenylphosphine oxide
