# Supplementary material for: Manganese-Substituted Polyoxometalates as Functional Mimics of Indole Dioxygenase Enzymes
Source: Inorg Chem. 2026 Jun 1;65(23):13276–82. doi: 10.1021/acs.inorgchem.6c02005 (PMC13273813; doi:10.1021/acs.inorgchem.6c02005)
Supplement: Supplementary file 1 [file ic6c02005_si_001.pdf]

Manganese Substituted Polyoxometalates as Functional Mimics of Indole Dioxygenase Enzymes

Adi Herman,[1] Raanan Carmieli,[2] and Ronny Neumann[1]*

1. Department of Molecular Chemistry and Materials Science, Weizmann Institute of Science, Rehovot 76100, Israel

2. Department of Chemical Research Support, Weizmann Institute of Science, Rehovot 76100, Israel
Email: ronny.neumann@weizmann.ac.il

Supplementary Information

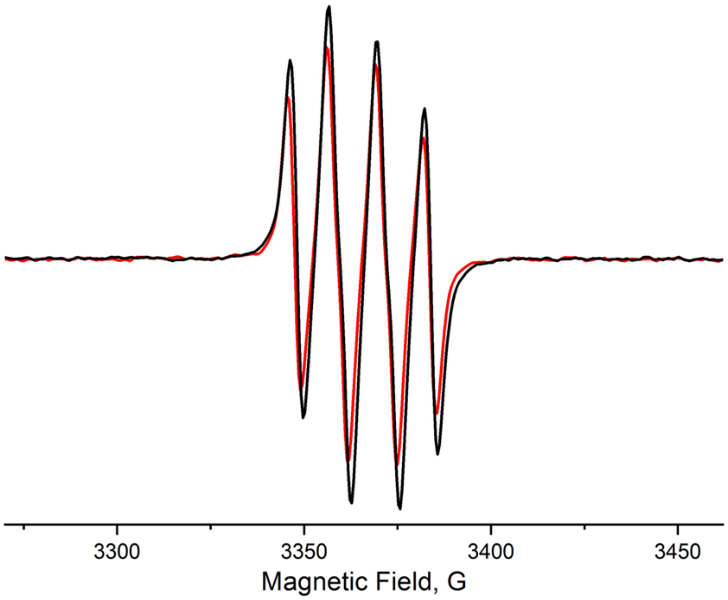

Figure S1: EPR spectra of $(THA)_{10}[SiMn_3(L_3)W_9O_{37}]$ in THF (red) and with (black) addition of DMSO after exposure to O_2. Conditions: 2mM $(THA)_{10}[SiMn_3(L_3)W_9O_{37}]$, 5 mg BMPO, 100 µL DMSO, 1.5 bar O_2, 1 h.

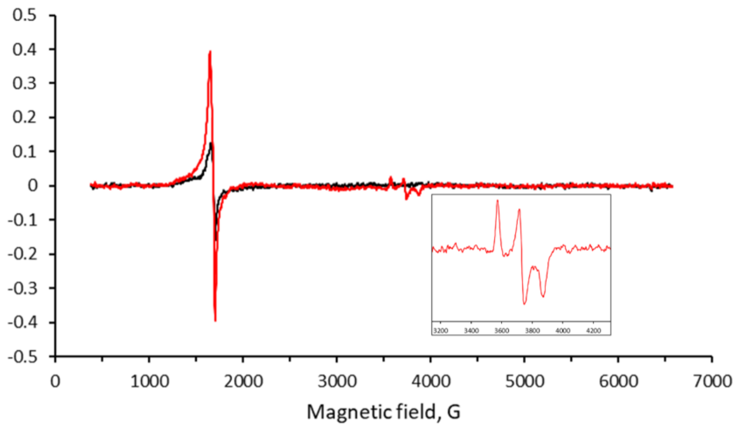

Figure S2: EPR spectrum (15 K) of 2mM $(THA)_{10}[SiFe_3(L_3)W_9O_{37}]$ in THF before (black) and after (red) exposure to air.

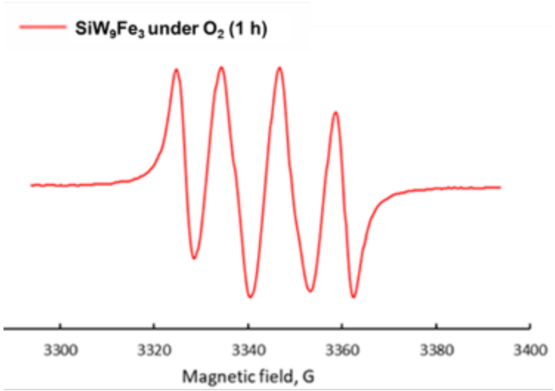

Figure S3: EPR spectrum of $(THA)_{10}[SiFe_3(L_3)W_9O_{37}]$ in THF after exposure to O_2 in the presence of BMPO. Conditions: 2 mM $(THA)_{10}[SiFe_3(L_3)W_9O_{37}]$, 5 mg/mL BMPO, 1.5 bar O_2, 1 h reaction.

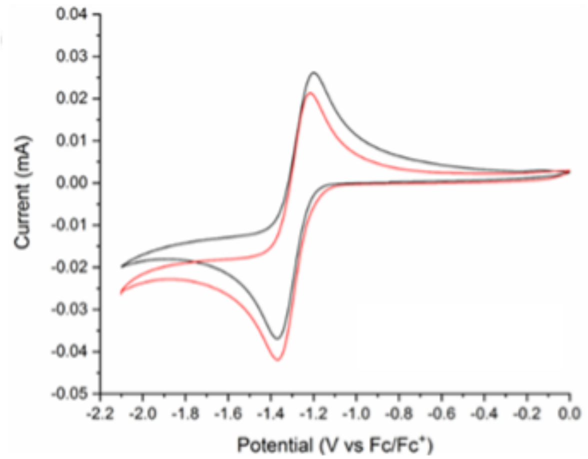

Figure S4: Cyclic voltammetry of O_2 in ACN with 0.1M $TBABF_4$ as supporting electrolyte (black line) and O_2 in ACN and in the presence $(THA)_{10}[SiMn_3(L_3)W_9O_{37}]$ (red line). Glassy carbon as working electrode, Pt wire as counter electrode and Fc/Fc^+ as reference electrode. scan rate: 50 mV/s.

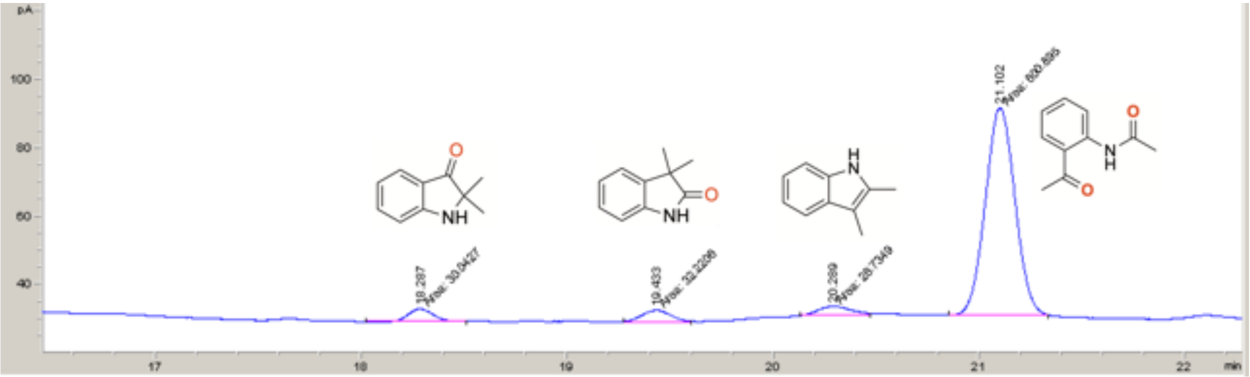

Figure S5. Representative GC-FID Chromatogram of Dioxygenase Reaction Showing Product Formation.

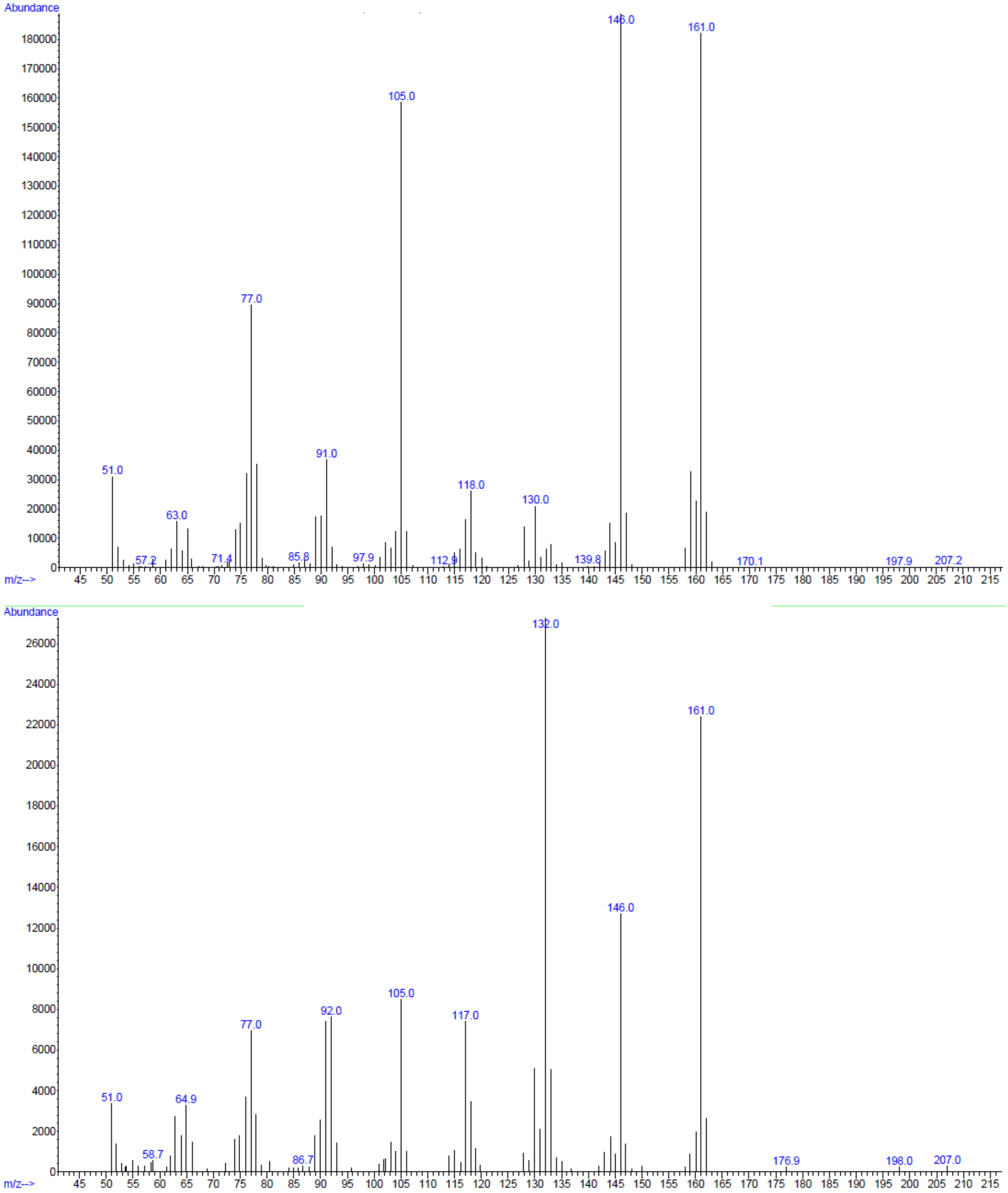

Figure S6. Mass Spectra from GC-MSD of 3,3-dimethylindolin-2-one (top) and 2,2-dimethylindolin-3-one (bottom).

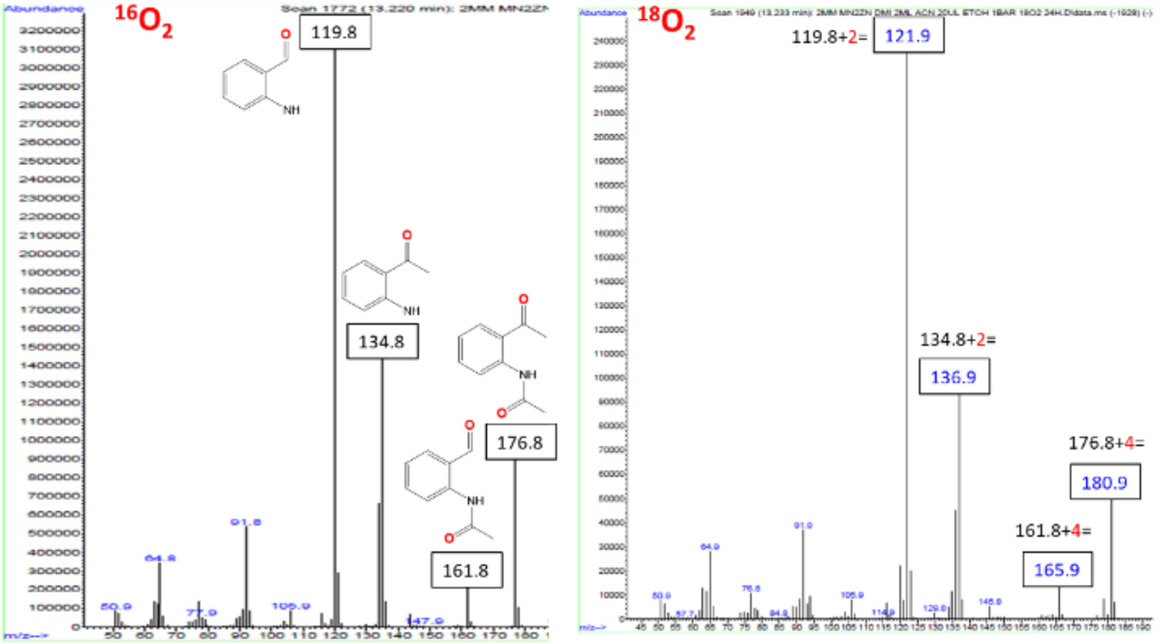

Figure S7. Mass spectra (from GC-MSD) of *N*-(2-acetylphenyl)acetamide in reactions under $^{16}O_2$ (left) and $^{18}O_2$ (right) carried out using {SiMnW$_{11}$} as catalyst.

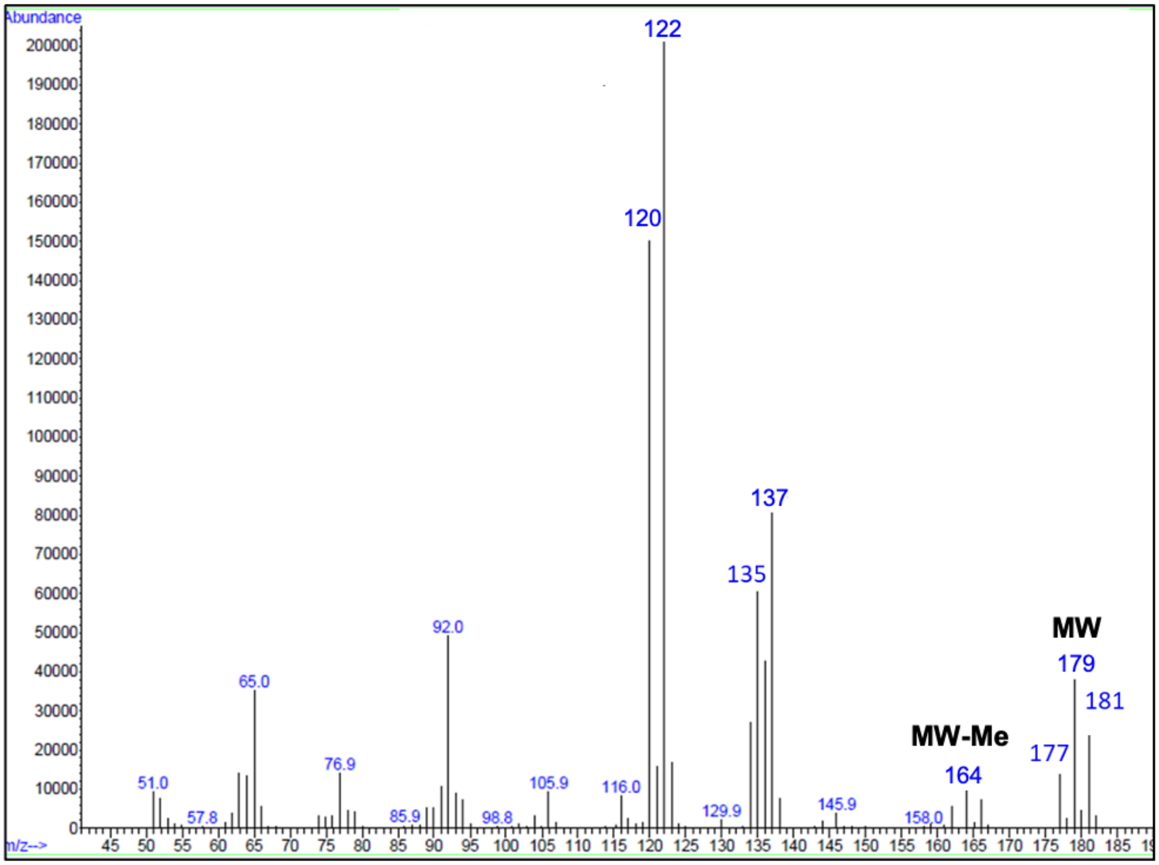

Figure S8: Mass spectrum (from GC-MSD) of 2-acetamidoacetophenone in a reaction using mixture of $^{16}O_2$ and $^{18}O_2$ carried out using {SiMnW$_{11}$} as catalyst.

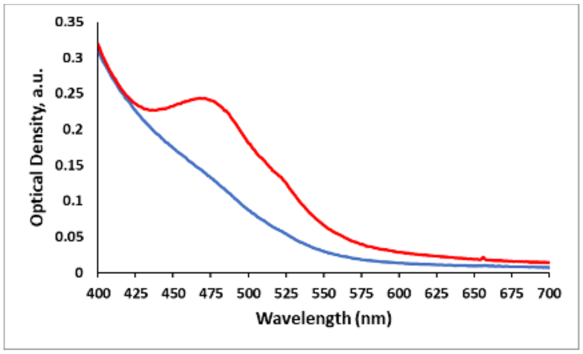

Figure S9. UV-vis spectra of 3 mM $(THA)_6[SiMn^{II}W_{11}O_{39}]$ in acetonitrile under N_2 (blue) and O_2 (red).

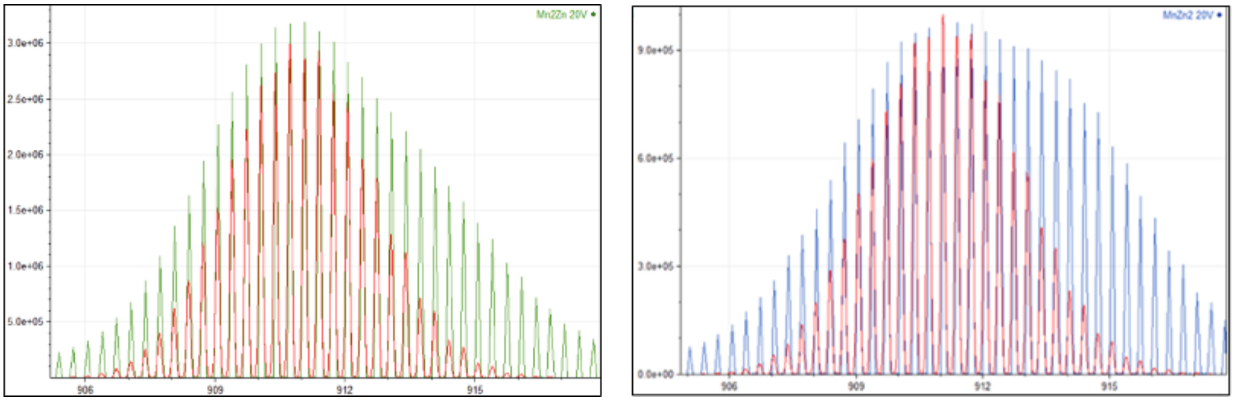

Figure S10. Left – Assigned peak from HR-ESI MS of $Cs_{10}[\beta\text{-}SiW_9O_{37}\{Mn_2Zn(H_2O)\}_3]$ $CsNaH_7[SiW_9O_{37}Mn_2Zn(CH_3CO_2)_2]$ (green) and simulation (red).

Right - Assigned peak from HR-ESI MS of $Cs_{10}[\beta\text{-}SiW_9O_{37}\{MnZn_2(H_2O)\}_3]$ $CsNaH_7[SiW_9O_{37}MnZn_2(CH_3CO_2)_2]^{3-}$ (blue) and simulation (red). Note that the shoulder on the right can be assigned to overlapping $CsNa_2H_6[SiW_9O_{37}MnZn_2(CH_3CO_2)_2]^{3-}$.

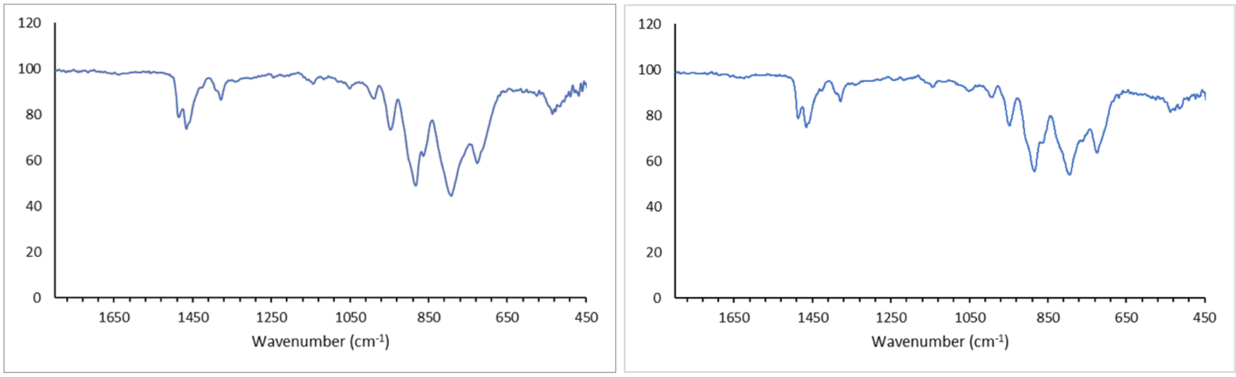

Figure S11. IR spectra of $Cs_{10}[\beta\text{-}SiW_9O_{37}\{Mn_2Zn(H_2O)\}_3]n$ (left) and $Cs_{10}[\beta\text{-}SiW_9O_{37}\{MnZn_2(H_2O)\}_3]$ (right)
